# Mindfulness, Compassion, and Self-Compassion as Moderator of Environmental Support on Competency in Mental Health Nursing

**DOI:** 10.1007/s42399-021-00904-5

**Published:** 2021-04-20

**Authors:** Fajar Rizal, Helen Egan, Michael Mantzios

**Affiliations:** 1 Department of Psychology, Birmingham City University, Birmingham, UK; 2 Department of Psychology, Faculty of Business, Law and Social Sciences, Birmingham City University, Room C307, The Curzon Building, 4 Cardigan St, Birmingham, B4 7BD UK

**Keywords:** Mental Health Nursing, Competency, Mindfulness, Self-compassion, Compassion, Environmental support

## Abstract

This research explored the established relationship between environmental support and competency for Mental Health Nurses, intending to investigate whether the tendency to display higher levels of mindfulness, compassion, and self-compassion might buffer the effect of a poor environment on competency. One questionnaire was comprised of five pre-developed questionnaires, which included all items examining environmental support, competency, mindfulness, compassion, and self-compassion. Mental Health Nurses (*n* = 103) were recruited from online forums and social media group pages in the UK. The result showed environmental support related positively to competency. Furthermore, the positive relationship of competency with environmental support was moderated when controlling for compassion but did not with mindfulness and self-compassion, although subscales showed some further interactions. When poor environmental support influences the competency of mental health professionals, compassion and mindfulness-based interactions may have the potential to uphold competency.

## Introduction

The National Health Service (NHS) encompasses a variety of healthcare professionals (HCPs) ranging from doctors, nurses, and students, all operating in different fields that involve working with a patient’s physical health, mental well-being, or both [1]. Fundamentally, the UK HCPs are expected to follow the “Leading Change, Adding Value” framework (i.e., care, compassion, competence, communication, courage, and commitment) expressing the standards that should help guide HCPs’ professionalism [2]. Accordingly, the intentions for such a framework are to enable enhanced compassionate care towards patients, stipulate a core structural plan for growth within the NHS, and provide reassurance to the general public and the health and mental health of HCPs [3–5]. As such, mental health nurses (MHNs) by and large engage directly with patients who have been diagnosed with a mental illness (e.g., schizophrenia or dissociative disorders) or individuals with a reduced level of overall well-being to specifically captivate effective therapeutic contact in emotionally intense situations [6]. Therefore, this practice aligns itself with the mandatory needs for compassion towards patients and builds on the culture for compassionate care within the mental health nursing profession [7].

The types of interactions between MHNs and patients are a primary aspect that is determining the workplace environment and environmental support that is predictive of effective care. One interesting aspect is the exposure of workplace aggression, ranging from verbal to targeted physical violence by individuals including patients, visitors, or other colleagues [8]; two types of aggression that will be briefly reviewed next. First, where physical violence occurs, MHNs are trained to adopt coercive practices including physical restraint and seclusions [9]. Despite the increased recognition of detriment from physically restraining patients for both patients and MHNs, restrictive practices (i.e., using medication or physical force; [10]) continue to be used within mental health nursing care. Muir-Cochrane et al. [11] investigated the views of using such practices within the mental health nursing community. Using nine focus groups with 44 MHNs across Australia, the research identified that both physical restraint and seclusion can cause feelings of fear and guilt in MHNs. The research highlighted that these emotions derive from the perceptions that MHNs feel guilty for using either medical or physical restraints on vulnerable individuals [12]. But they also reported a feeling of fear as if this practice was eliminated, they may lose the power to protect themselves and others due to the physical, mental, and legal ramifications of these practices and their consequences [13]. Therefore, MHNs may further experience cognitive dissonance (i.e., a state of having inconsistent thoughts, feelings, beliefs, or attitudes; [14]), especially relating to staff balancing the need for the ward safety, while at the same time providing person-centred care [8].

Second, another significant aspect of workplace aggression involves verbal violence, including verbal abuse and bullying. Tonso et al. [15] conducted quantitative research on a sample of 1600 mental health service employees in Australia and investigated the exposure to violence in the workplace and related psychological health outcomes. The research found a significant number of MHN professionals experiencing verbal abuse and bullying, followed by physical violence. The study further identified that one in three victims of violence rated themselves as being in psychological distress. Typical forms of abuse on MHNs generally involve an attack on competence, threats, and physical insults, and this can at times create an ambience of hostility, which can affect the patient-nurse interaction [16]. Further evidence derived by Edward et al.’s [17] systematic review highlighted that verbal abuse experienced by MHNs can increase the burden on workers, for example, with increased emotional labour (i.e., the effort one places to suppress own emotions to care for others effectively while also caring for oneself [18], and increase burnout and attrition [19]. Consequently, Edward et al. [17] recommend that clinical practices should consider the learning environment for students and the relationship between students and supervisors, and consider a supportive work environment involving decision-making and the provision of ongoing professional development opportunities that facilitate the development of emotional intelligence and resilience [17, 20].

Barriers to providing compassionate care include emotional dissonance [21] and burnout [22–25], and these have all been linked to occupational stress (i.e., the pressure between the person and the working environment due to the responsibilities, conditions, environment, or other difficulties of the workplace; [26]). Roche et al. [27] investigated the quality of nursing practices concerning individual competency (i.e., a nurse’s ability and willingness to engage in a therapeutic relationship), and found that experienced nurses (e.g., those who perceived themselves to be supported and competent) were more likely to express the willingness to engage with their patients effectively. Yet, this can only be possible if environmental factors (i.e., the quality of nurse training, opportunities to participate in hospital affairs, and clinical supervision) are available.

Current and past findings are further supported by research that highlighted several organisational difficulties contributing to uncompassionate care, including understaffing [25], high workload [26], and insufficient funding and resources [27]. Insufficient funding, resources, and understaffing can have profound effects on the ability of MHNs to work effectively. An imbalance of workload, high nurse-patient ratios, and inadequate physical resources can negatively impact nurses’ ability to work and are detrimental to self-care practices at work [4, 28]. These difficulties may act as obstructions to compassion and competence in caring for patients effectively, while the current lack of improving the healthcare environment to enhance competent care may be targeted by improving compassion, and selfcompassion within the MHN profession.

Generally, HCPs prioritise their patients’ needs over their own needs for effective support and care. Compassion, a fundamental element of caring, has been identified as an experience of empathising with others’ suffering, which motivates individuals to alleviate pain either physically, mentally, or emotionally by understanding the perspective and emotions of another individual [32]. Research within the MHN community has demonstrated that compassion is important for healthcare providers as a prerequisite to provide a high degree of care to patients over time. Compassion fatigue—a term used to describe [33] the emotion when a person experiences the negative effect of working in a psychologically distressing environment—can result in limiting an MHNs professional ability to feel compassion for others, and often leads to other psychological and physiological effects such as burnout and attrition [34]. Kim and Lee [35] investigated the association between compassion, competence, burnout, missed nursing care, professional quality of life, and quality of life amongst clinical nurses. Findings suggest that as compassion and competency decreased, missed nursing care and burnout increased. However, when compassion increased, professional quality of life and general quality of life amongst nurses also increases, ultimately improving quality of care, patient experience, and patient safety. The concept of compassion is well documented in the healthcare literature, but Barron et al. [36] proposed that there has been limited attention in the mental health nursing profession. They further propose that MHNs could benefit from training to facilitate their understanding of compassionate practices and improving their competency.

Similarly, self-compassion can be defined as an experience of recognising one’s suffering (e.g., perceived failures, competency, or general suffering) and responding by aiming to alleviate the suffering [37]. Past research suggested this through three main components including self-kindness (vs. self-criticism; [38]), common humanity (vs. isolation; [39]), and mindfulness (vs. over-identification; [40]). Indeed, low self-compassion amongst MHNs associates negatively with self-criticism, which has been found to positively relate to symptoms, such as depression and anxiety; impacting one’s competency and life satisfaction [41]. McGowan [42] investigated the properties of self-compassion and inclusion to reduce burnout amongst MHPs. The findings highlighted that self-compassion is significantly associated with lower burnout rates, along with higher scores of wellbeing and compassion for others, although inclusion mediated the relationship between self-compassion and burnout, which suggest that supportive environments can explain the relationship between self-compassion and burnout rates [38]. However, self-compassion is a component of mindfulness which is described as being attentive to the present moment and not being judgmental to oneself when experiencing stressful environments (e.g., complex situations in daily tasks; [43, 44]).

Ruiz-Fernández et al. [45] conducted a systematic review investigating the effectiveness of a mindfulness-based intervention on stress, self-compassion, and mindfulness in MHNs. As nurses encounter negative experiences while caring for patients, families, or interacting with other healthcare teams, Penque [46] further established the effectiveness of having a higher trait score in mindfulness and suggested that mindfulness may improve nurses’ wellbeing by adopting a healthier working environment and promote positive reactions to stress. Considering the evidence established on mindfulness-based reduction techniques, it is not surprising that mindfulness practices often reduce stress, improve teamwork, and in turn, improve competency [47]. Nevertheless, there is an apparent neglect of compassion, self-compassion, and mindfulness research in promoting well-being amongst health professionals. Mills et al. [48] mentioned that for nurses who are faced with suffering (such as occupational stress), self-compassion is essential for their self-care (e.g., taking frequent breaks) and overall wellbeing, which is also linked to an ability to be compassionate for others and can improve overall competency. [49], as cited in ([4], p. 3) suggest that “the need to be kind to oneself (psychological health) can lead to unkind health behaviours (physiological health)”, for example, being kind to oneself in the short term by taking a smoking break has a negative physiological consequence (e.g., cardiovascular diseases; [50]; see also [51]),

This research explores the relationship between environmental support and competency further and additionally explores how such relationships can change with elements of compassion, self-compassion, and mindfulness traits to understand the positive healthcare practices amongst students and qualified MHNs. Therefore, it was hypothesised that there is a positive relationship between low self-reported environmental support and low competency scores, and such interaction can be moderated through the presence of compassion, self-compassion, and mindfulness.

## Methods

### Participants

Two-hundred and twenty-six mental health nurses from the health care community responded to the invitation to take part in this research. Preceding analysis, the data were screened for outliers, normality, and missing data. After deleting excessively missing data, one-hundred and three participants remained (38 males; 65 females) with a mean age of 30.73 (*SD* = 10.01). Through snowball sampling [52], mental health nurses were recruited via online social media group pages and forums. No incentives were used to recruit participants and participation was voluntary. To achieve a medium effect size, a minimum of 77 participants were required [53].

### Material

#### Demographic Questionnaire

Participants’ background information such as gender, age, profession, and experience, as well as a previous and current diagnosis of ill mental health, was explored as a method of analysing based on recruiting a healthy mental health nursing population.

#### Nurse Professional Competence Scale (NPCS; [54])

The original NPCS contains 88 items to measure self-reported competence amongst nursing students and registered nurses, though this research adopted the short form consisting of 35 items due to administration timescale. The principal components of the scale consisted of six factors including nursing care (5 items); value-based nursing care (5 items); medical and technical care (6 items); care pedagogics (5 items); documentation and administration of nursing care (8 items); and development, leadership, and organisation of nursing care (6 items). Example items include “Meet patient’s basic physical needs” measured nursing care; “Contribute to a holistic view of the patient” measured value-based nursing care; “Independently administer prescriptions” measured medical and technical care; “Inform and educate groups of patients and relatives” measured care pedagogics; “Lead and develop health staff teams” measured documentation and administration of nursing care; and “Teach, supervise and assess students” measured development, leadership, and organisation of nursing care. The scale calculation scores are measured on a 5-point Likert scale which ranged from 1 (to a very low degree) to 5 (to a very high degree). The present study produced a Cronbach’s alpha score of .94 for the overall score.

#### Learning Environment, Supervision, and Teacher Scale (CLES+T; [55, 56])

CLES+T consists of 34 items, designed to measure self-reported quality assessment and evaluation of nursing education. The subject matter of clinical learning environments is covered by five sub-dimensions including pedagogical atmosphere (9 items), leadership style of the ward (4 items), nursing care on the ward (4 items), supervisory relationship (8 items), and role of nurse teacher (9 items). Example items include “The staff was easy to approach” measures pedagogical atmosphere, “The effort of individual employees was appreciated” measures leadership style of the ward, “The ward’s nursing philosophy was clearly defined” measured nursing care on the ward, “I felt that I received individual supervision” measured supervisory relationship, and “The nurse teacher helped me to reduce the theory-practice gap” measured the role of nurse teacher. The scale calculation scores were measured on a 5-point Likert scale which ranged from 1 (fully disagree) to 5 (fully agree). The present study produced a Cronbach’s alpha score of .93.

#### Five-Facet Mindful Questionnaire (15-FFMQ; [57])

Similar to the original 35 item FFMQ [58], the questionnaire consists of 15 items measuring five facets including non-reactivity to inner experiences, observing, act awareness, describing, and non-judging of inner experiences. Example items include the following: “When I take a shower or a bath, I stay alert to the sensations of water on my body” and “I’m good at finding words to describe my feelings” which measured non-react and describe mindfulness, while items such as “I don’t pay attention to what I’m doing because I’m daydreaming, worrying, or otherwise distracted” and “I believe some of my thoughts are abnormal or bad and I shouldn’t think that way” measured act aware and non-judge components of mindfulness. Additionally, for example, an item such as “When I have distressing thoughts or images, I take a step back” measured non-react. The calculation scores consisted of responses measured on a 5-point Likert scale which ranged from 1 (*never or very rarely true*) to 5 (*very often or always true*); however, some items were scored in reverse (i.e., 1=5, 2=4, 3=3, 4=2 and 5=1). The present study produced a Cronbach’s alpha score of .75.

#### Sussex-Oxford Compassion for Self and Others (SOCS; [59])

SOCS consists of a total of 40 items measuring compassion for others (20 items) and compassion for self (20 items). Example items include “I recognise when other people are feeling distressed without them having to tell me”, “I notice when others are feeling distressed”, and “I recognise signs of suffering in others” measured the compassion for others, while items such as “I understand that everyone experiences suffering at some point in their lives”, “I notice when I’m feeling distressed”, and “When I’m upset, I do my best to take care of myself” measured the compassion for the self. The scores consisted of responses measured on a 5-point Likert scale which ranged from 1 (not true at all) to 5 (always true). The present study produced a Cronbach’s alpha score of .93.

### Procedure

Ethical approval was obtained from the ethical board within the university and this study followed ethical practices following the British Psychological Society. Through Qualtrics (version June 2020; [60]), individuals were recruited via forums and social media groups pages (e.g., Facebook, Twitter, Instagram, and Linked-in) using a hyperlink. The general information page and consent form was administered first. The participants signed the consent form by creating a unique candidate number with indicated voluntary participation. The participants began the research by answering CLES+T, followed by NPCS, then the 15-FFMQ and concluding with SOCS. The debrief form indicated the end of the research. Participants only attend one testing session, which lasted approximately 30–45 minutes.

### Statistical Analysis

All statistical analysis was conducted using IBM SPSS v24. Data were initially explored through bivariate correlation. Data were entered into SPSS 17.0. Descriptive statistics (mean and standard deviation) were used for continuous variables and numbers were applied for categorical variables. Moderation effects were interpreted by using PROCESS v3.4 (Model 1) with a bootstrap sample of 5000 where variables were cantered to their means [61]. Simple effects coefficients were computed for the three values including the subscales of the moderator (i.e., 1 *SD* below the mean, at the mean, and 1 *SD* above the mean). For all analyses, *p*-values ≤ .05 were considered statistically significant; nevertheless, the bootstrapping procedure and use of bias-corrected confidence intervals (*CI*) were determined to attribute the statistical significance of the moderator [62].

## Results

Initially, a dependence bivariate correlation (i.e., Pearson’s) was conducted, and findings suggested a positive correlation between compassion and self-compassion traits to environmental support on competence, but mindfulness did not (Table 1).

**Table 1:** Pearson’s bivariate correlation summary for variables of interest (*n* = 103)

Variables	NPCS	CLES+T	FFMQ-15	SOCS-O	SOCS-S	Mean	SD
NPCS	-					103.77	17.11
CLES+T	.476**	-				109.06	17.60
FFMQ-15	.160	.292**	-			47.49	8.22
SOCS-O	.463**	.277**	-.080	-		79.20	13.97
SOCS-S	.348**	.287**	.123	.326**	-	70.07	11.92

Correlation is significant *(*p* < .05) **(*p* <.01).

*NPCS* Nurse professional competence scale, *CLES+T* Learning Environment, Supervision and Teacher Scale, *FFMQ-15* 15-item Five-Facet Mindfulness Questionnaire, *SOCS-O* Sussex-Oxford Compassion for Others, *SOCS-S* Sussex-Oxford Compassion for Self

A simple linear regression was calculated to predict competency, based on self-reported environmental support (*b* = 51.58, *t*(1) = 5.42, *p* =.000). A significant regression was found, *F* (1,102) = 29.52, *p* =.000, with an r^2^ of .23. Significant positive association were observed between environmental support and competency to mindfulness (*b* = 49.97, *t*(2) = 4.36, *p* = .000); compassion (*b* = 28.74, *t*(2) = 2.79, *p* = .000) and self-compassion (*b=* 36.19, *t*(2) = 3.29, *p =* .000).

Further explorations to testing mindfulness, compassion, and self-compassion as potential moderators for the relationships between environmental support and competence. Firstly, mindfulness overall model suggests significant variance, *F*(3,99) = 18.18, *p* = .000, *r*^2^ = .253, but failed at the highest unconditional order interaction, *F*(1, 99) = 2.29, *p* = .133, Δ*R*^2^ = .026. However, under the average conditional interaction, mindfulness (*b* = .536, *t*(99) = 2.95, *p* = .000) suggests some relationship between environmental support and competency. Further analysis of the slopes for environmental support predicting competency at each level of mindfulness score indicated some interactions on the subscales (Table 2). Specifically, acting with awareness appears to significantly shift the relationship and be a significant moderator, *F*(1, 99) = 14.96, *p* = .000, Δ*R*^2^= .014. Results indicated that the positive significant relationship between environmental support and competency becomes significant as mindful subscales scores increase but not when mindfulness total score increases.

**Table 2:** Conditional effects of the subscales of mindfulness on the relationship between the environment and competency (*n* = 103)

		β	*p*	95% *CI*	
				Lower	Upper
Observe	-1 SD	-2.50	.046	.006	.631
	At the mean	.000	.000	.288	.674
	+1 SD	2.50	.000	.348	.938
Describe	-1 SD	-1.89	.000	.249	.657
	At the mean	.000	.000	.252	.706
	+1 SD	1.89	.003	.171	.838
Act with awareness	-1 SD	-2.54	.023	.057	.759
	At the mean	.000	.000	.330	.675
	+1 SD	2.54	.000	.360	.833
Non-judge	-1 SD	-3.02	.266	-.324	1.16
	At the mean	.000	.000	.273	.702
	+1 SD	3.02	.014	.116	.993
Non-react	-1 SD	-2.64	.004	.124	.628
	At the mean	.000	.000	.336	.661
	+1 SD	2.64	.000	.414	.829

Note: *SD* standard deviation, *CI* confidence intervals, *p* significance level, *β* regression coefficient

Secondly, testing self-compassion overall model as potential moderator for the relationship between environmental support and competence found to be significant, *F*(3, 99) = 15.09, *p* = .000, *r*^2^ = .276, but self-compassion failed at the highest unconditional order interaction, *F*(3, 99) = .314, *p* = .576, Δ*R*^2^ = .002. Under the average conditional interaction, self-compassion, *b* = .410, *t*(99) = 4.932 *p* = .000, suggests some relationship between environmental support and competency. Further analysis of the slopes for environmental support predicting competency at each level of self-compassion score indicated some interactions on the subscales (Table 3). Specifically, understanding the universality of suffering appears to significantly shift the relationship and be a significant moderator, *F*(1,99) = 37.98, *p* = .000, Δ*R*^2^ = .417. Results indicated that the positive significant relationship between environmental support and competency becomes significant as self-compassion subscales scores increase but not when self-compassion total score increases.

**Table 3:** Conditional effects of the subscales of self-compassion on the relationship between the environment and competency (*n* = 103)

		β	*p*	95% *CI*	
				Lower	Upper
Recognising suffering	-1 SD	-2.98	.002	.175	.757
	At the mean	.000	.000	.239	.622
	+1 SD	2.98	.002	.148	.641
Understanding the universality of suffering	-1 SD	-3.06	.229	-.090	.474
	At the mean	.000	.000	.212	.507
	+1 SD	3.06	.000	.430	.726
Feeling for the person suffering	-1 SD	-3.10	.000	.229	.681
	At the mean	.000	.000	.246	.635
	+1 SD	3.10	.001	.174	.678
Tolerating uncomfortable feeling	-1 SD	-3.55	.000	.284	.720
	At the mean	.000	.000	.256	.649
	+1 SD	3.55	.006	.118	.689
Acting or being motivated to alleviate suffering for self	-1 SD	-3.25	.002	.150	.674
	At the mean	.000	.000	.253	.634
	+1 SD	3.25	.000	.252	.699

Note: *SD* standard deviation, *CI* confidence intervals, *p* significance level, *β* regression coefficient

Testing compassion overall model as a potential moderator for the relationship between environmental support and competency found to be significant, *F*(1,99) = .33.16, *p* = .000, Δ*R*^2^ = .383, and significant at the highest unconditional order interaction, *F*(3, 99) = 8.00, *p* = .006, Δ*R*^2^ = .03, suggesting some interaction. Slopes for environmental support predicting competency at each conditional effect of compassion was analysed. For low compassion score, environmental support, *b* = .135, *t*(99) = 1.09, *p* = .277, suggests that there is no relationship between environmental support and competency. Average compassion score, *b* = .319, *t*(99) = 3.70, *p* = .000, suggests that there is a significant relationship increasing competency. High compassion scores, *b* = .503, *t*(99) = 5.58, *p* = .000, suggests a high significant relationship increasing competency. Further analysis for testing the slopes for environmental support predicting competency at each level of compassion score indicated the interactions on the subscales (Table 4). Specifically, acting or being motivated to alleviate suffering for others appears to significantly shift the relationship and be a significant moderator, *F*(1,99) = 27.88, *p* = .000, Δ*R*^2^ = .376. Results indicated that the positive significant relationship between environmental support and competency becomes significant as compassion total scores increases.

**Table 4:** Conditional effects of the subscales of compassion on the relationship between the environment and competency

		β	*p*	95% *CI*	
				Lower	Upper
Recognising others	-1 SD	-2.76	.004	.104	.539
	At the mean	.000	.000	.220	.568
	+1 SD	2.76	.000	.280	.652
Understanding the universality of suffering	-1 SD	-3.38	.108	-.054	.537
	At the mean	.000	.000	.220	.559
	+1 SD	3.38	.000	.364	.713
Feeling for the person suffering	-1 SD	-3.29	.115	-.042	.383
	At the mean	.000	.000	.161	.523
	+1 SD	3.29	.000	.263	.753
Tolerating uncomfortable feeling	-1 SD	-3.28	.052	-.002	.409
	At the mean	.000	.000	.173	.521
	+1 SD	3.28	.000	.269	.710
Acting or being motivated to alleviate suffering for others	-1 SD	-3.22	.192	-.093	.458
	At the mean	.000	.000	.164	.516
	+1 SD	3.22	.000	.316	.678

Note: *SD* standard deviation, *CI* confidence intervals, *p* significance level, *β* regression coefficient

## Discussion

The present study was aimed to identify the relationship between environmental support and competency and investigate the corresponding moderating role of compassion, self-compassion, and mindfulness. Often nurses encounter negative experiences while caring for patients or interacting with other healthcare professionals, having a supportive environment, where individuals feel a sense of inclusion and support from colleagues and superiors can help foster a healthier working environment. Findings indicated that environmental support correlated positively with competency suggesting that as environmental support decreases, competency also decreases. This result confirms that supportive environments can provide preparedness to practise and help MHNs to develop a deepened knowledge, which improves teamwork and positively impacts on overall clinical learning [17, 20].

Contrary to the hypothesised associations, the interaction between mindfulness and environmental support did not influence self-reported competency. However, moderation effects between the subscales of mindfulness and environmental support imply that there are elements of mindfulness that can contribute to overall competency [46, 47]. Specifically, acting with awareness is associated with higher competency. Therefore, future studies should explore current findings further by considering the subscales of mindfulness. Additionally, the moderating effects between self-compassion and environmental support did not influence self-reported competency. However, the moderating effects between the subscales of self-compassion and environmental support indicate that there are elements of self-compassion contributing to a higher competency [41, 42]. Specifically, understanding the universality of suffering is associated with higher competency. Thus, future studies should explore the current findings further by considering the subscales of self-compassion.

Finally, the present study showed that the interaction between compassion and environmental support influenced self-reported competency. Moderation effects of compassion indicated that individuals with high compassion traits reported higher levels of competency than those who experience low levels of compassion when observing the relationship between competency and environmental support. This result confirms earlier findings that underline the strong positive associations between compassion and environmental support on competency [33, 35]. Furthermore, it is important to note that the subscales of compassion, specifically acting or being motivated to alleviate suffering for others are associated with higher overall competency.

While higher scores in mindfulness, self-compassion, and compassion are desirable for holistic health, wellbeing, and competency (i.e., compassionate care and self-care) within nurses, there is limited evidence and partial confirmation on mindfulness, self-compassion, and compassion positively influencing mental health nursing students and professionals. The limited available evidence also contributes to a lack of use of mindful-based interventions on improving health and wellbeing. Therefore, there is a pressing need to find holistic support for MHN within the UK, which takes into account environmental support and addresses the psychological difficulties and barriers to achieve optimal competency. Given the complex funding and resources provided for the NHS, this is a considerable challenge. This research proposes the potential of mindfulness, self-compassion, and compassion to create an affordable focus on environmental support to improve health and wellbeing, for both patients and MHNs. Undoubtedly, compassion, self-compassion, and mindfulness need to be practised regularly, which can be challenging in a workplace that is sometimes lacking basic support, and alternative and more practical interventions can be trialled and implemented with minimal cost and minimal commitment [63–66].

There are two notable limitations in the present research. First, the design of the research was cross-sectional; therefore, no firm conclusion could be drawn on the interactional design between the constructs, although this was a necessary first step as a method to assess MHN support and competency. Closely related, use of mindfulness and compassion-based trait need to be investigated in experimental settings and/or longitudinal designs, to demonstrate credible changes in improving competency. Second, the data were collected during a pandemic (i.e., COVID-19), and thus, data could be comparably different when experiencing “normal” timeframes, where the world is functioning without the heightened fear of death and disease. The empirical results reported herein should be considered in light of these limitations, but future research should aim to incorporate this information alongside more detailed and current accounts of personal and professional life.

In conclusion, the current study shows that higher scores on environmental support are associated with higher competency scores within mental health nursing. These findings also provided evidence that compassion may be a useful trait in supporting MHN’s competency. Additionally, subscales of mindfulness and self-compassion may also moderate the interaction between environmental support and competency. Further research is needed to corroborate these results and build controlled empirical studies that test the efficacy of mindfulness and compassion traits for improving the learning environments within MHN and to better equip those who struggle to be attentive, compassionate, and caring to their patients.

## Data Availability

Data will be made available by the first author upon request.
